# When models choose metrics: Hidden geometry in computational biology

**DOI:** 10.1371/journal.pcbi.1014789

**Published:** 2026-09-17

**Authors:** Dillion M. Fox

**Affiliations:** AIML, GSK, Collegeville, Pennsylvania, United States of America; Johns Hopkins University, UNITED STATES OF AMERICA

## Abstract

Computational biology often turns biological measurements into fitted models, embeddings, or summaries and then compares them with a distance chosen largely by convention. That choice can shape the scientific question being answered, because different distances emphasize different assumptions about noise, variation, and biological similarity. This Perspective describes the hidden geometry that can arise when a probabilistic model is specified carefully enough to support local metric structure. For regular parametric models, the Fisher information defines a natural local metric, making distance choice part of model specification rather than a separate plotting or clustering preference. Rather than replacing familiar heuristics, information geometry helps explain when common transformations, such as variance stabilization for RNA-seq, approximate a model-implied distance; when dependence or latent nuisance structure limits that approximation; and when misspecification makes empirical, nonparametric, or sensitivity-based comparisons more appropriate. The goal is a clearer, more explicit practice of metric choice.

## 1 The hidden geometry of parametric models

Consider a typical analysis in computational biology. A researcher sequences gut microbiome samples from a treatment and control group, fits a compositional count model, and then asks the central scientific question: how different are these communities? To answer, the researcher may choose Bray-Curtis dissimilarity, UniFrac, Jensen-Shannon divergence, or another metric supported by the field’s software and literature [1–3]. These are often defensible choices. The geometric point is narrower: the analysis usually treats the distributional model and the downstream metric as independent decisions, when the first should inform the second.

A practical rule of thumb is to ask what objects the distance is actually comparing. If the objects are fitted probability distributions in a regular parametric family, and the fitted model is supported by diagnostics or domain assumptions, the natural starting point is the geometry implied by that model. If the fitted model is only a rough surrogate, the metric should be treated as part of a sensitivity analysis across model-based, transformed Euclidean, local covariance estimates, and empirical distances. If the objects being compared are empirical distributions, latent coordinates, or learned embeddings, the metric should be justified for those objects rather than borrowed automatically from the measurement model.

To see why, consider the univariate Gaussian, parameterized by mean μ and standard deviation σ. Plotting two such distributions by their parameters invites a straight Euclidean line with squared distance $d\mu^{2}+d\sigma^{2}$, but this ruler ignores statistical distinguishability. A one-unit mean shift carries more weight when σ is small and distributions are localized than when σ is large and they overlap heavily. The geometry is driven by overlap of probability distributions, not merely by parameters drawn on a page.

When you parameterize a family of probability distributions, you are not merely specifying a likelihood; you are selecting a subset of the space of all distributions and equipping it with a geometry. The negative binomial distributions form a smooth two-dimensional surface; the Dirichlet distributions over a compositional vector form a higher-dimensional curved manifold. These are not metaphors but mathematical objects with well-defined structure, and that structure carries a natural notion of distance without any further decision on your part.

The relevant mathematics is information geometry, a field developed over the past five decades by Amari and Nagaoka [4], Chentsov, and others. The central object is the *statistical manifold*: a smooth space whose points are probability distributions, so that moving in this space corresponds to changing the distribution, represented locally by model parameters θ. For a regular, identifiable model with fixed support and nonsingular Fisher information, the Fisher information matrix F(θ)—the expected outer product of the score function, measuring how rapidly the distribution changes as parameters vary—serves as a local Riemannian metric tensor: a rule for turning very small parameter changes into statistical lengths and angles. Geodesic distances are obtained by finding paths with shortest integrated local length through this model space.

What makes this more than a mathematical nicety is a theorem due to Chentsov [5], with related treatments and extensions by [6] and [7]. In finite sample spaces, under its formal invariance assumptions, Chentsov’s theorem says that the Fisher information metric is the *unique* Riemannian metric, up to a scale factor, that is invariant under sufficient statistics; related extensions exist for exponential-family settings. Here, “sufficient” means preserving all model-relevant information about the parameters: if two data representations contain the same evidence about the modeled generative process, the metric assigns the same intrinsic local geometry. Like any Riemannian metric, the Fisher metric can be written in different coordinates, but the intrinsic lengths it defines do not depend on the arbitrary parameterization. Within these regular model settings, the Fisher-Rao metric therefore provides a principled way to derive a local metric from the chosen assumptions. The practical conclusion is not that Fisher-Rao geometry should always be used, but that every metric should be accountable to the object and assumptions it is meant to compare.

## 2 Model misspecification and graceful degradation

Information geometry encodes the geometry of the *chosen* distribution. But what happens when that distribution is a poor or misleading representation of the data?

If the data-generating process in nature is not well represented by a negative binomial or Dirichlet model—or if any fitted predictive model imposes independence assumptions that the biology violates—the Fisher-Rao metric measures distances on the specified model manifold, which may not describe the biological question. Under these conditions of misspecification, the sensitivity of the Fisher metric becomes a liability. Because it depends on the variance and covariance structure implied by the parameterized model, forcing an incorrect distributional assumption onto empirical data can distort downstream distances.

To understand why, consider the paradoxes that arise under common forms of misspecification. When a complex empirical distribution is fit with an overly simple parametric model, distance metrics can tell different stories about the data. These cases are illustrated schematically in S1 Fig, which contrasts Euclidean parameter distance, Fisher-Rao distance, and one-dimensional Wasserstein distance.

If we fit a single Gaussian to a bimodal distribution, the resulting parametric mean and variance might match those of a distribution with a different shape. Under this “matched moments” paradox, distances between the fitted Gaussian summaries can collapse even though distances between the empirical distributions would not.

The Fisher metric is additionally vulnerable to the “variance mirage.” Because Fisher-Rao geometry scales distance inversely with model variance, tail contamination in an otherwise clean distribution can inflate the fitted variance and shrink the implied distance between mean shifts. Nonparametric distances such as Wasserstein distance can remain sensitive to distributional shape that a fitted parametric summary discards, but they introduce their own choices: the ground metric, scaling, sampling noise, dimensionality, and robustness to outliers all matter. Finally, simple Euclidean distance on the parameters—though it lacks Fisher-Rao invariance and remains vulnerable to matched-moment failures—can be a useful robustness check, since it does not inherit the model’s full variance scaling.

A compact landscape of metric choices is therefore more useful than a single ranking. Fisher-Rao or other model-implied geometries are strongest when the comparison is between fitted distributions in a trusted regular model family. Transformed Euclidean distances such as VST-Euclidean are computationally cheap approximations when the transform stabilizes the relevant model variance, but they inherit the transform’s marginal or factorization assumptions. Domain-structured distances such as UniFrac can be appropriate when the biological question explicitly involves phylogenetic turnover rather than only count uncertainty or likelihood geometry. Optimal transport or Wasserstein distances compare empirical distributions and can preserve distributional shape under misspecification, but in high-dimensional single-cell or multi-omics settings they can be computationally expensive, sensitive to ground metric and scaling, unstable for sparse count matrices, and harder to interpret. Learned-representation embeddings can bypass an explicit parametric geometry by learning task-aligned coordinates, but they shift the burden to the training objective, data coverage, calibration, and validation; they relocate metric assumptions rather than eliminate them.

This suggests a practical decision framework:

- **Parametric assumptions are well supported:** Use the model-implied information geometry, or an approximation to it, as the default local metric.
- **Model diagnostics fail:** Use nonparametric methods, empirical covariance estimates, or bootstrap-based stability checks to assess whether conclusions depend on the parametric geometry.
- **Intermediate cases:** Treat metric choice as a sensitivity analysis. The Fisher metric may expose the model’s natural geometry, but simple Euclidean or nonparametric distances may degrade more gracefully when the model only partly fits the biology.

## 3 Classical anchors

### Microbiome and compositional data

Microbiome datasets show why this is not merely a mathematical preference. Aitchison geometry [8] captures relative-abundance structure by applying log-ratio transformations to the simplex. In contrast, multinomial, Dirichlet, or Dirichlet-multinomial models emphasize compositional count uncertainty and the discrete count noise of the sequencing instrument [9,10]. UniFrac adds a different kind of structure: phylogenetic tree geometry, in which branch lengths encode relatedness among taxa and community differences can be measured by how abundance shifts across lineages [2]. A Fisher-Rao comparison under a Dirichlet-multinomial or related count model asks how distinguishable two fitted abundance distributions are under that sampling model; a tree-aware distance asks how much community mass turns over across related taxa. None of Aitchison, Fisher-Rao, or UniFrac is universally right; each answers a different model/question pair, and the better choice depends on whether the biological question concerns relative-abundance, compositional count uncertainty, or phylogenetic turnover.

### RNA-seq and the negative binomial gap

In differential expression analysis, the negative binomial (NB) distribution is widely used to model overdispersed read counts [11]. To visualize sample relationships via PCA or UMAP, standard pipelines instruct users to apply a variance-stabilizing transformation (VST) and compute Euclidean distances.

**Fig 1:**
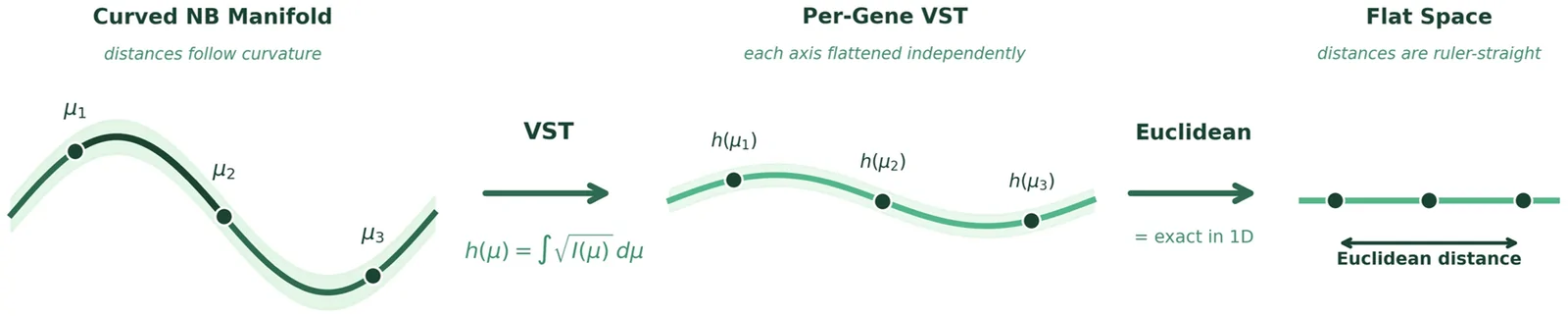
The variance-stabilizing transformation (VST) as a Fisher-Rao isometry. The VST is the integral of the square root of the Fisher information, mapping the 1D negative binomial manifold to an arc-length parameterization. This mapping is an exact isometry for a single gene with fixed-dispersion. The geometric tension arises in multi-gene settings where co-expressed modules and gene-gene dependence break the product-manifold or marginal-factorization assumption that justifies applying the per-gene VST independently.

This connection can also be understood through heteroscedasticity. In RNA-seq, variance changes with the mean; for a negative binomial model, $Var(Y|\mu )=\mu +\alpha \mu^{2}$. A VST corrects this mean-dependent noise so that equal Euclidean steps carry more comparable statistical weight. Information geometry gives the same correction a metric interpretation: in one dimension with fixed-dispersion, Fisher information scales local distance by the inverse variance, $I(\mu )=1/(\mu +\alpha \mu^{2})$. The VST is therefore the arc-length coordinate obtained by integrating the square root of this Fisher information, making it an isometry—a distance-preserving reparameterization—for the one-gene fixed-dispersion model (Fig 1). Applying Euclidean distance after the VST is not a rejection of information geometry; it is an implicit Fisher proxy.

The limitation appears when this one-gene approximation is applied feature-by-feature to correlated expression. If an immune-activation module shifts many interferon-response genes together while a cell-cycle module shifts another block, per-gene VST-Euclidean distance treats repeated correlated shifts as independent coordinates. A joint model would encode those dependencies through covariance, off-diagonal Fisher information, or latent variables, so the per-feature distance can overcount coordinated changes and miss module-level structure. Practitioners can check this approximation with gene-correlation or module summaries, residual PCA, bootstrap stability of clustering or ordination, and comparisons to covariance-aware or empirical distances.

## 4 Where does the geometry live?

The practical question is not only whether a model has a Fisher metric, but which object is compared and what a downstream algorithm consumes. Clustering and ordination usually operate on observed sample profiles, learned embeddings, transformed feature matrices, or pairwise dissimilarity matrices, whereas the Fisher information is defined locally on the parameter manifold of a fitted model. A claim such as “use the Fisher-Rao distance” is therefore incomplete until the observations have been mapped to model parameters, and the local metric converted into the pairwise distances or coordinates the algorithm uses.

For count data, two cells or bulk RNA-seq samples with vectors $y_{a}$ and $y_{b}$ must first be mapped, under a chosen negative binomial model and choices about size factors, dispersion, and covariates, to fitted parameters or estimated distributions. The Fisher information gives local costs on that model manifold; a clustering algorithm needs a finite pairwise distance, obtained through a geodesic, local quadratic approximation, or model-motivated transform such as VST. S1 Text works this through numerically, from counts to a pairwise distance.

This local-to-global step is where many practical choices enter. The distinction matters because an observed count vector, an inferred embedding, and a fitted distribution are different objects. Comparing output distributions asks how similar the distributions the model induces are; comparing intermediate embeddings asks how similar points are in a learned-representation space, whose geometry is not automatically that of the measurement model.

Sometimes the biological object of interest is latent. A cell state, antigenic coordinate, or ecological gradient may be inferred through nuisance variables such as batch, sequencing depth, serum potency, or sampling effort that affect the measurement but are not the biological target. The Fisher information then lives on the full parameter space of the measurement model, and a metric on the biological coordinates is obtained only after marginalizing or projecting away the nuisance directions. Treating the latent coordinates as if they inherited a flat Euclidean geometry can hide this extra modeling choice.

Two common cases show why the distinction matters. In a well-specified RNA-seq or single-cell model, library size, sequencing depth, plate, or batch may be included explicitly as nuisance parameters. The biologically relevant metric is then derived after those directions are marginalized out of the Fisher information, not by measuring distance along technical axes. This is not automatic post-processing but a modeling choice about which coordinates belong to the biological object and which to the measurement process.

By contrast, if batch effects or other technical confounders are unmodeled latent causes of variation, the original measurement model’s geometry may be invalid for the intended biological comparison. Distances can then treat sequencing run, preparation, or potency artifacts as if they were intrinsic biological movement. Nuisance-aware geometry then cannot be recovered by a downstream coordinate correction; the model, comparison object, or sensitivity analysis has to change.

## 5 Conclusion

The choice of a distance metric is inextricably linked to the structure of the underlying statistical model, yet recognizing this hidden geometry does not mean enforcing it in every pipeline: the curvature of the Fisher metric is only as reliable as the parametric assumptions that generate it. When a model is regular, identifiable, and locally nonsingular, the Fisher information defines the natural local metric for its manifold; when it is misspecified, that failure should be visible as a modeling failure, not hidden inside a convenient distance function. Adopting an information-geometric perspective does not invalidate the flat-space heuristics that degrade gracefully in practice; it makes explicit what we trade away when we use them.

### Implications for metric choice

- **Check your parametric assumptions:** Before committing to a parametric metric such as Fisher-Rao, validate whether the statistical model fits your data; if it is severely violated, use cheap safeguards first, such as bootstrap stability, residual diagnostics, sensitivity across baseline metrics, or local covariance estimates, and reserve heavier nonparametric methods such as Optimal Transport for when distributional shape is central.
- **Compare metric families deliberately:** Treat model-implied, transformed Euclidean, domain-structured, empirical, and learned-representation distances as alternative assumptions about the biological object.
- **Use the Fisher information as a diagnostic:** Examine the local metric a model implies before choosing a downstream distance.
- **Know your isometries:** If you use a heuristic metric for speed, identify which parametric geometry it approximates, such as VST as a 1D NB isometry, and where it breaks down, such as gene-gene covariance.
- **Consider comparing distributions:** When a model provides probabilistic outputs, ask whether to compare summary representations or the output distributions they induce.

## Supporting information

S1 Fig Schematic divergence of distance metrics under parametric misspecification. **(A)** Under ideal specification, Euclidean parameter distance measures flat metric separation. **(B)** Under ideal specification, Fisher measures discriminability, shrinking the space when variance is high. **(C)** A schematic 5% tail contamination inflates parametric variance, causing the Fisher metric to underestimate separation relative to the uncontaminated state. **(D)** Severe misspecification (Bimodal vs Normal) causes both parametric metrics to collapse to zero, masking structural differences. **(E)** Oppositely skewed distributions with aligned modes but different means and tail directions remain distinguishable to all three metrics; their Gaussian summaries retain the mean shift but discard the asymmetric shape. **(F)** A Uniform distribution compared to a Normal distribution with identical moments illustrates the insensitivity of parametric metrics to higher-order shape changes.
(PDF)

S1 Text Glossaries and derivations. Definitions and worked derivations for the Gaussian example, the misspecification schematic, and the negative binomial variance-stabilizing transformation.
(PDF)

S1 Code Python source for the misspecification schematic. Code and instructions for regenerating S1 Fig.
(PY)
