# Supplementary material for: When models choose metrics: Hidden geometry in computational biology
Source: PLoS Comput Biol. 2026 Sep 17;22(9):e1014789. doi: 10.1371/journal.pcbi.1014789 (PMC13585257; doi:10.1371/journal.pcbi.1014789)
Supplement: Supplementary file 1 [file pcbi.1014789.s001.pdf]

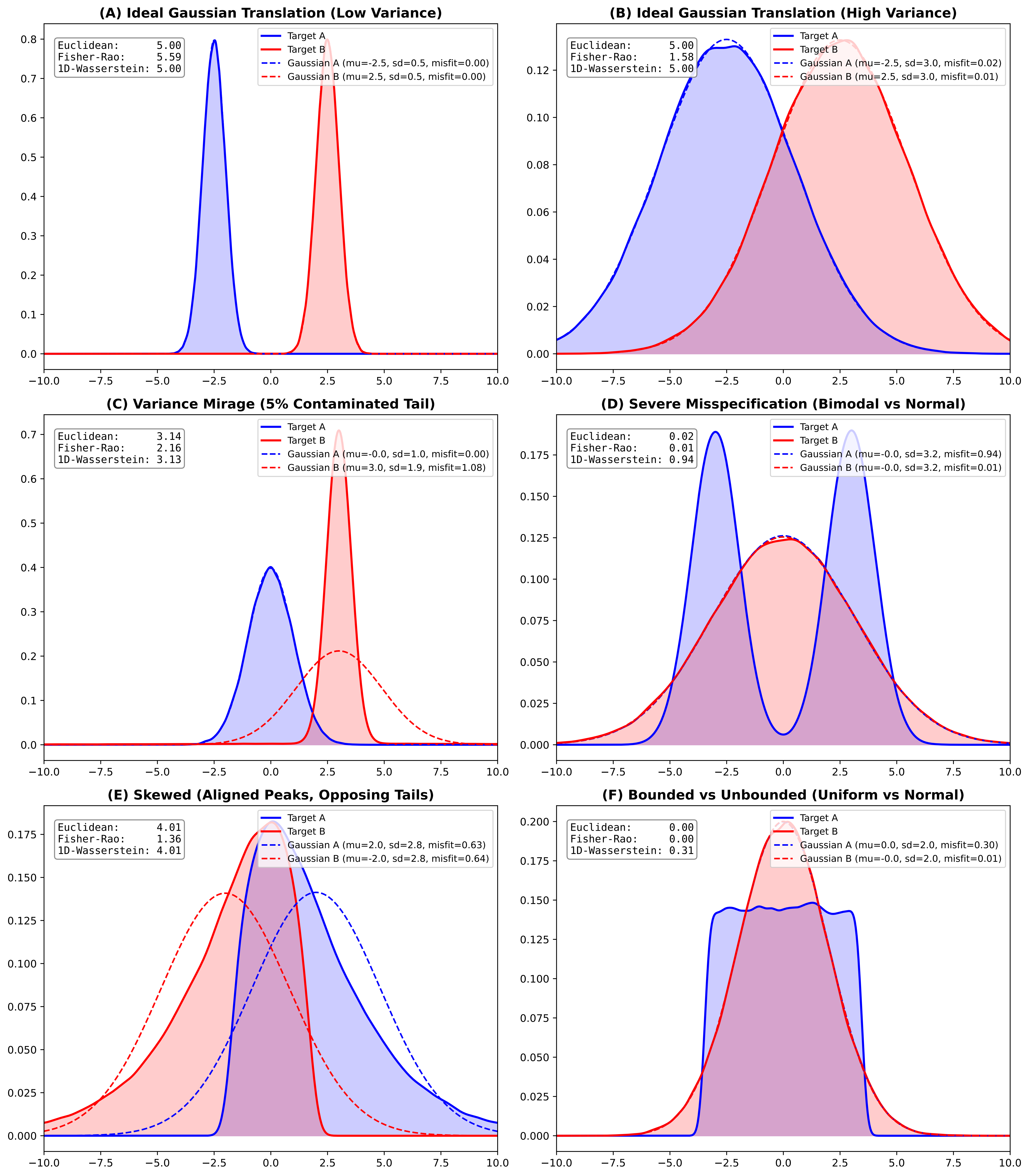

(A) Ideal Gaussian Translation (Low Variance)
Euclidean: 5.00
Fisher-Rao: 5.59
1D-Wasserstein: 5.00
Target A
Target B
Gaussian A (mu=-2.5, sd=0.5, misfit=0.00)
Gaussian B (mu=2.5, sd=0.5, misfit=0.00)
(B) Ideal Gaussian Translation (High Variance)
Euclidean: 5.00
Fisher-Rao: 1.58
1D-Wasserstein: 5.00
Target A
Target B
Gaussian A (mu=-2.5, sd=3.0, misfit=0.02)
Gaussian B (mu=2.5, sd=3.0, misfit=0.01)
(C) Variance Mirage (5% Contaminated Tail)
Euclidean: 3.14
Fisher-Rao: 2.16
1D-Wasserstein: 3.13
Target A
Target B
Gaussian A (mu=-0.0, sd=1.0, misfit=0.00)
Gaussian B (mu=3.0, sd=1.9, misfit=1.08)
(D) Severe Misspecification (Bimodal vs Normal)
Euclidean: 0.02
Fisher-Rao: 0.01
1D-Wasserstein: 0.94
Target A
Target B
Gaussian A (mu=-0.0, sd=3.2, misfit=0.94)
Gaussian B (mu=-0.0, sd=3.2, misfit=0.01)
(E) Skewed (Aligned Peaks, Opposing Tails)
Euclidean: 4.01
Fisher-Rao: 1.36
1D-Wasserstein: 4.01
Target A
Target B
Gaussian A (mu=2.0, sd=2.8, misfit=0.63)
Gaussian B (mu=-2.0, sd=2.8, misfit=0.64)
(F) Bounded vs Unbounded (Uniform vs Normal)
Euclidean: 0.00
Fisher-Rao: 0.00
1D-Wasserstein: 0.31
Target A
Target B
Gaussian A (mu=0.0, sd=2.0, misfit=0.30)
Gaussian B (mu=-0.0, sd=2.0, misfit=0.01)
