# Supplementary material for: When models choose metrics: Hidden geometry in computational biology
Source: PLoS Comput Biol. 2026 Sep 17;22(9):e1014789. doi: 10.1371/journal.pcbi.1014789 (PMC13585257; doi:10.1371/journal.pcbi.1014789)
Supplement: Supplementary file 2 [file pcbi.1014789.s002.pdf]

S1 Text: Glossaries and Derivations for When Models Choose Metrics: Hidden Geometry in Computational Biology

Dillion M. Fox[1]
[1]*AIML, GSK, Collegeville, Pennsylvania, United States of America*
dillion.m.fox@gsk.com

S1 Text: Glossaries and Derivations

This supporting file provides selected glossary material, schematic mathematical examples, and derivations for the PLOS Computational Biology Perspective "When Models Choose Metrics: Hidden Geometry in Computational Biology."

References

Amari, S.-I. and Nagaoka, H. (2000). *Methods of Information Geometry.* American Mathematical Society.

Chentsov, N. N. (1982). *Statistical Decision Rules and Optimal Inference.* American Mathematical Society. (Original work published 1972).

Love, M. I., Huber, W., and Anders, S. (2014). Moderated estimation of fold change and dispersion for RNA-seq data with DESeq2. *Genome Biology*, 15(12):550.

Appendix

A A Conceptual Glossary of Information Geometry & Statistics

Amari and Nagaoka. Shun-ichi Amari and Hiroshi Nagaoka [Amari and Nagaoka, 2000] formalized the mathematical bridge between statistics, information theory, and differential geometry. Their work established that regular families of probability distributions can often be treated as differentiable manifolds, where the Fisher information matrix acts as the local metric tensor.

Chentsov's theorem. A foundational uniqueness result in information geometry showing that, under formal regularity and invariance conditions, the Fisher information metric is the unique Riemannian metric up to a scaling factor that is invariant under sufficient statistics [Chentsov, 1982]. In this Perspective, the theorem motivates deriving local metrics from regular likelihood models rather than treating metric choice as independent of model choice.

Compositional distributions. Probability models or data representations for vectors that describe parts of a whole, such as relative abundances or counts conditioned on a fixed total. The sum constraint couples the components, so the appropriate geometry depends on whether the analysis treats the data as compositional coordinates, multinomial or Dirichlet observations, or another model-based representation.

Dirichlet distributions. A family of continuous multivariate probability distributions parameterized by a vector of positive reals, commonly used to model compositional fractions that sum to one. The Dirichlet family has its own Fisher geometry, so flat Euclidean distances on raw coordinates should not be assumed to reflect the model-implied geometry.

Fisher-Rao distance. The geodesic distance between two probability distributions on a regular statistical manifold, calculated using the Fisher information metric. It provides a parameterization-invariant measure of local statistical distinguishability within the chosen model.

Heteroscedasticity. A statistical setting in which the variance of observations changes across the range of the mean or covariates. In RNA-seq, negative binomial count models are heteroscedastic because $\mathrm{Var}(Y \mid \mu) = \mu + \alpha\mu^2$. Variance-stabilizing transformations correct this mean-dependent noise; information geometry interprets the same correction as a local metric rescaling by Fisher information.

Jensen-Shannon divergence. A symmetrized and smoothed version of the Kullback-Leibler divergence. For regular statistical manifolds, its second-order local expansion is proportional to the Fisher information metric; the square root of Jensen-Shannon divergence is the metric version commonly used for finite comparisons.

Likelihood models with nuisance parameters. Statistical models where the parameters of interest (e.g., biological state) must be estimated alongside necessary but uninteresting parameters (e.g., sequencing depth or batch effects). In information geometry, the Fisher metric is first defined on the full regular parameter space; a metric on the biological coordinates then requires an additional operation such as conditioning, marginalization, or projection, depending on the inferential target.

Negative binomial distributions. A discrete probability distribution widely used to model overdispersed count data, such as RNA-seq read counts, where the variance exceeds the mean. Once a parameterization is fixed, the model has a corresponding Fisher information; in the fixed-dispersion one-dimensional case used below, that information sets the local scale for changes in the mean.

Variance Stabilizing Transformations (VST). A mathematical transformation applied to reduce mean-dependent variance, commonly used in transcriptomics (e.g., DESeq2). In the idealized one-dimensional model with fixed dispersion, the VST corresponds to the integral of the square root of the Fisher information, making it an arc-length coordinate for the fixed-dispersion Fisher-Rao metric.

B A Conceptual Glossary of Biological Terminology

Aitchison geometry. A geometry for compositional data—vectors of relative abundances constrained to sum to one—built from log-ratio transformations of the simplex, so that differences are measured between ratios of components rather than raw proportions. It captures relative-abundance structure, but it is a coordinate choice on the simplex rather than a distance derived from a specific count-generating likelihood, so it answers a different question than a Dirichlet-multinomial Fisher-Rao comparison.

Bray-Curtis dissimilarity. A popular dissimilarity for quantifying differences in species abundance profiles. It is useful in many ecological settings but is not derived from a single generative likelihood; this makes it a practical contrast case for model-derived geometries.

Size factor. A per-sample normalization constant in RNA-seq that rescales counts to account for differences in sequencing depth or library size, so that gene-level means are comparable across samples. In a negative binomial model the size factor enters as a known offset on the mean; it is a nuisance quantity that must be fixed or estimated before a model-implied distance is computed.

UniFrac. A phylogenetic distance between microbial communities that weights differences in taxon abundance by the branch lengths separating taxa on a reference tree, so that shifts among closely related lineages count for less than shifts among distant ones. Unlike a Dirichlet-multinomial Fisher-Rao comparison, which measures how distinguishable two fitted abundance distributions are under a count model, UniFrac injects externally supplied tree geometry, making it appropriate when the biological question concerns phylogenetic turnover.

C The Geometry of the Univariate Gaussian

To ground these abstract geometrical concepts, it is instructive to derive the Fisher information metric for a familiar family of probability distributions: the univariate Gaussian, parameterized by its mean μ and standard deviation σ.

The probability density function is given by:

$$p(x \mid \mu, \sigma) = \frac{1}{\sqrt{2\pi\sigma^2}} \exp\left(-\frac{(x-\mu)^2}{2\sigma^2}\right) \tag{1}$$

To define a geometric distance between two nearby distributions, we measure how distinguishable they are given a small change in their parameters θ. The first-order fractional change in the probability density caused by an infinitesimal parameter shift $d\theta$ is $\frac{\nabla_\theta p(x|\theta)^\top d\theta}{p(x|\theta)}$. By the chain rule, this is $\nabla_\theta \log p(x \mid \theta)^\top d\theta$. The score therefore provides the local object whose expected squared size defines the Fisher information.

For the Gaussian, the log-likelihood is:

$$\log p(x \mid \mu, \sigma) = -\tfrac{1}{2}\log(2\pi) - \log\sigma - \frac{(x-\mu)^2}{2\sigma^2} \tag{2}$$

To understand why the metric can be written using second derivatives, it helps to recall that the log-likelihood measures how compatible a parameter value is with the data-generating distribution. Its gradient is the score, and under standard regularity conditions the expected outer product of

the score is equal to the negative expected Hessian. This expected curvature of the log-likelihood defines the Fisher information matrix, which serves as the local metric tensor on the statistical manifold.

To capture the expected variance of this relative change, we define the Fisher information matrix $\mathcal{I}(\theta)$ for the parameter vector $\theta = (\mu, \sigma)$ as the negative expectation of this Hessian matrix (the second derivatives of the log-likelihood):

$$\mathcal{I}_{ij} = -\mathbb{E}\left[\frac{\partial^2 \log p}{\partial\theta_i\,\partial\theta_j}\right] \tag{3}$$

Taking the second partial derivatives yields:

1. With respect to the mean: $\dfrac{\partial^2 \log p}{\partial\mu^2} = -\dfrac{1}{\sigma^2}$
2. With respect to the standard deviation: $\dfrac{\partial^2 \log p}{\partial\sigma^2} = \dfrac{1}{\sigma^2} - \dfrac{3(x-\mu)^2}{\sigma^4}$
3. The cross derivative: $\dfrac{\partial^2 \log p}{\partial\mu\,\partial\sigma} = -\dfrac{2(x-\mu)}{\sigma^3}$

We evaluate the expectation $\mathbb{E}$ over the random variable $x \sim \mathcal{N}(\mu, \sigma^2)$. Noting that $\mathbb{E}[x-\mu] = 0$ and $\mathbb{E}[(x-\mu)^2] = \sigma^2$, the matrix simplifies considerably:

$$\mathcal{I}(\mu, \sigma) = \begin{pmatrix} \frac{1}{\sigma^2} & 0 \\ 0 & \frac{2}{\sigma^2} \end{pmatrix} \tag{4}$$

This matrix serves as the metric tensor g_{ij} for the statistical manifold.

If we naively construct a 2D coordinate system using μ and σ as axes and map two Gaussian distributions to this plane, it is immediately tempting to draw a straight line between the points and calculate the Euclidean distance, $dl^2 = d\mu^2 + d\sigma^2$.

However, using the Fisher information derived above gives the Fisher-Rao line element, which defines the infinitesimal distance between two nearby Gaussian distributions within this model:

$$dl^2 = \frac{d\mu^2}{\sigma^2} + 2\,\frac{d\sigma^2}{\sigma^2} \tag{5}$$

This metric differs from the naive Euclidean assumption. The $1/\sigma^2$ scaling factors make local distance reflect statistical distinguishability under the Gaussian model. A one-unit shift in the mean ($\Delta\mu = 1$) does not carry the same informational weight when the variance is large compared to when it is near zero. If one considers two highly localized distributions (small σ), a small shift in their means can make them much easier to distinguish. Conversely, if two distributions have large variance, they overlap heavily and are harder to distinguish, so the metric shrinks the local distance between them. The geometry is driven by the specified probability model, not merely by the parameters drawn on a page.

D Parametric Misspecification Paradoxes

S1 Fig provides a schematic illustration of how parametric distance metrics, such as the Fisher-Rao metric or Euclidean distance on fitted parameters, can behave under idealized forms of distributional misspecification. In each panel, a Gaussian approximation (dashed line) is compared with

a conceptual target distribution (solid line). The panel is intended as a mathematical schematic rather than an empirical study result.

The schematic contrasts the parametric Euclidean distance ($\sqrt{(\mu_1 - \mu_2)^2 + (\sigma_1 - \sigma_2)^2}$), the Riemannian Fisher-Rao distance, and the nonparametric 1D Wasserstein distance (Earth Mover's Distance). The displayed misfit value is the Wasserstein distance between the conceptual target distribution and the Gaussian approximation.

E The geometry of the negative binomial and the VST

To explicitly demonstrate how the Variance Stabilizing Transformation (VST) acts as a geometric isometry, we can examine the Fisher Information for the negative binomial (NB) distribution, which forms the foundation of RNA-seq differential expression models like DESeq2 [Love et al., 2014].

In these models, the read counts for a single gene are modeled as a negative binomial distribution parameterized by its mean μ and a dispersion parameter α, such that the variance is given by $\sigma^2 = \mu + \alpha\mu^2$. Standard pipelines typically estimate α across all samples and then treat it as a fixed, known constant for each gene. This reduces the parameter space to a one-dimensional statistical manifold parameterized solely by the mean μ.

For this one-dimensional fixed-dispersion manifold, the Fisher information scalar for the mean parameter is:

$$I(\mu) = \frac{1}{\mu + \alpha\mu^2} \tag{6}$$

To see this directly, write $r = 1/\alpha$ and use the negative binomial parameterization with fixed size r and mean μ. For an observed count k, the score with respect to μ is:

$$\frac{\partial \ell}{\partial \mu} = \frac{r(k - \mu)}{\mu(r + \mu)} \tag{7}$$

Since $\mathrm{Var}(K) = \mu + \mu^2/r$, the Fisher information is:

$$I(\mu) = \mathbb{E}\left[\left(\frac{\partial \ell}{\partial \mu}\right)^2\right] = \frac{r^2}{\mu^2(r + \mu)^2}\left(\mu + \frac{\mu^2}{r}\right) = \frac{r}{\mu(r + \mu)} = \frac{1}{\mu + \alpha\mu^2} \tag{8}$$

The Riemannian line element (the infinitesimal distance dl on this manifold) is therefore defined by:

$$dl^2 = I(\mu)\, d\mu^2 = \frac{1}{\mu + \alpha\mu^2}\, d\mu^2 \tag{9}$$

The Fisher information is the squared local scale factor in the metric. The infinitesimal arc length is therefore:

$$dl = \sqrt{I(\mu)}\, |d\mu| = \frac{|d\mu|}{\sqrt{\mu + \alpha\mu^2}} \tag{10}$$

To compute the distance between two negative binomial distributions with means μ_1 and μ_2 under this fixed-dispersion model, we integrate this line element, not the Fisher information itself:

$$d_{\mathrm{FR}}(\mu_1, \mu_2) = \left| \int_{\mu_1}^{\mu_2} \sqrt{I(\mu)}\, d\mu \right| \tag{11}$$

This arc-length coordinate is the geometric form of a Variance Stabilizing Transformation for the fixed-dispersion mean parameter. If we define a coordinate transformation $z = f(\mu)$ such that:

$$f(\mu) = \int \sqrt{I(\mu)}\, d\mu = \int \frac{1}{\sqrt{\mu + \alpha\mu^2}}\, d\mu \tag{12}$$

The resulting transformed coordinate z evaluates to:

$$z = f(\mu) = \frac{2}{\sqrt{\alpha}} \operatorname{arcsinh}(\sqrt{\alpha\mu}) \tag{13}$$

This shifts coordinates to an arc-length parameterization on the 1D manifold. In this new z-coordinate system, the metric coefficient is exactly 1 everywhere:

$$g_{zz} = I(\mu)\left(\frac{d\mu}{dz}\right)^2 = \frac{I(\mu)}{(f'(\mu))^2} = 1 \tag{14}$$

Thus, Euclidean distance between VST-transformed mean coordinates is not a rejection of information geometry; for a single negative binomial mean parameter with fixed dispersion, it is the closed-form Fisher-Rao arc length. Applying the transform to observed counts in practical pipelines is an approximation to this model-level statement. For multiple genes, the same model-level statement holds under an independent product-manifold approximation with fixed per-gene dispersions, including the case where each gene has its own fixed dispersion and corresponding transform. The important caveats are dependence, nuisance-parameter estimation, size-factor uncertainty, dispersion uncertainty, and broader model mismatch. The product of 1D independent manifolds is intrinsically flat after the per-gene arc-length transform, but treating genes as independent when they are biologically correlated can distort downstream distances.

F From a local metric to a pairwise distance: a worked count example

A natural practical question is how the local Fisher information on a model manifold becomes the single number a clustering or ordination algorithm consumes. This appendix answers that concretely for count data, reusing the fixed-dispersion negative binomial arc-length coordinate derived in Section E rather than re-deriving it; we write K_{gc} and α_g for the count and fixed dispersion of gene g in sample c. To keep the exact construction on one common statistical manifold, the worked example conditions on equal effective size factors across the two samples.

Scope. This realizes the VST/arc-length route to a pairwise distance under an independent negative binomial product model; the geodesic and local-quadratic routes, and covariance-aware, optimal-transport, or learned-embedding distances, answer related but different questions.

Recipe.

1. **Fix the model and its known constants.** Model each gene g as negative binomial with mean μ_{gc} in sample c, in the spirit of DESeq2's fixed-dispersion modeling: a dispersion α_g is estimated across samples and then treated as known. The example also conditions on a common effective library size. (DESeq2's implemented VST uses estimated size factors and a fitted mean–dispersion trend; the per-gene closed form here is the idealized fixed-α, common-depth case.)

2. **Map counts to a mean estimate.** With one observation per sample, the count $\hat{\mu}_{gc} = K_{gc}$ on this common scale is a plug-in point estimate of the mean—unbiased but high-variance—not a distribution fitted from replicates. The distance below therefore compares plug-in fitted models and does not propagate this estimation uncertainty. With unequal size factors, the

normalized-count variance has a sample-dependent linear term, so depth must be modeled explicitly as a nuisance quantity; the common transform below is no longer an exact shared isometry merely because counts were divided by their size factors.

3. **Convert each mean to the Fisher arc-length coordinate.** Apply the transform from Section E, $z_{gc} = \frac{2}{\sqrt{\alpha_g}} \operatorname{arcsinh}\sqrt{\alpha_g\, \hat{\mu}_{gc}}$. Because $dz/d\mu = \sqrt{I(\mu)}$, each gene's one-dimensional manifold is mapped isometrically to a coordinate in which its metric is exactly Euclidean.

4. **Form the pairwise distance.** If the genes are modeled independently with fixed per-gene dispersion, the statistical manifold is a Riemannian product and its geodesic distance is the Euclidean distance in the stacked coordinates,

$$D_{\mathrm{VST}}(a,b) = \sqrt{\sum_g \left(z_{ga} - z_{gb}\right)^2}. \tag{15}$$

   This is *exactly* the Fisher-Rao geodesic distance of the independent product model; it *approximates* the joint Fisher-Rao distance only to the extent that independence and fixed dispersion hold.

5. **Assemble the object the algorithm consumes.** Because D_{VST} is ordinary Euclidean distance in z, the stacked coordinate vector is itself an embedding: one can pass the samples-by-genes z matrix directly to any Euclidean-metric method (k-means, PCA, UMAP, neighbor graphs), or, for methods that ingest a dissimilarity, repeat step 4 over all pairs to build the matrix $\{D_{\mathrm{VST}}(a,b)\}$. Either way it is this z-geometry, not the local Fisher information itself, that the algorithm sees.

A two-sample example. Take two RNA-seq samples a and b measured on three genes, with a common fixed dispersion $\alpha_g = 0.1$ and equal effective size factors ($s_a = s_b = 1$, so $\hat{\mu}_{gc} = K_{gc}$). Here $2/\sqrt{\alpha_g} \approx 6.325$.

Gene	K_a	K_b	z_a	z_b	Δz
g_1 (low expression)	10	40	5.57	9.13	3.56
g_2 (high expression)	1000	1200	18.96	19.54	0.57
g_3 (low expression)	50	55	9.77	10.04	0.28

The model-implied distance is $D_{\mathrm{VST}}(a,b) = \sqrt{3.56^2 + 0.57^2 + 0.28^2} \approx 3.61$. For comparison, the naive Euclidean distance on raw counts is $\sqrt{30^2 + 200^2 + 5^2} \approx 202.3$.

Why the two numbers tell different stories. The metrics disagree about *what* makes the two samples different. Of the *squared* distance, raw-count Euclidean is $\approx 98\%$ attributable to g_2, the highly expressed gene with a 200-count gap, whereas D_{VST} is $\approx 97\%$ attributable to g_1, the $10 \to 40$ shift at low expression. Neither is simply right or wrong: raw Euclidean answers "how large is the absolute change in counts," while D_{VST} answers "how statistically distinguishable are the fitted means under the negative binomial model." Under that model the accounting is deterministic: at $\mu \approx 1000$ the variance $\mu + \alpha\mu^2 \approx 1.0 \times 10^5$ makes a 200-count gap statistically minor, whereas at $\mu \approx 10$ the variance is only ≈ 20, so a fourfold mean shift is highly distinguishable. Locally $dz = d\mu/\sqrt{\mathrm{Var}}$, so equal steps in z are equal increments of statistical distinguishability (a cumulative, saturating quantity, not a linear error rate). One honest tension: the low-count regime that dominates D_{VST} is also where the single-observation $\hat{\mu}$ is noisiest and where DESeq2-style

shrinkage would most change z, so with real data one would stabilize these estimates before trusting the reweighting.

Where it breaks. The equality in step 4 presumes genes are modeled independently, so the per-gene Fisher information is diagonal by construction. Co-expression does not add off-diagonal terms to that per-gene model—it means the per-gene model is the wrong one: a *joint* model would encode the dependence through covariance, off-diagonal Fisher information, or shared latent variables, and its geodesic distance need not equal the stacked-z Euclidean distance, so D_{VST} can overcount coordinated shifts across a module. The diagnostics recommended in the main text—correlation or module summaries, residual PCA after VST, bootstrap stability of clustering, and comparison against covariance-aware or empirical distances—test whether this approximation materially distorts the resulting distances. Simple stabilizing transforms such as log-CPM give broadly similar rankings here because they too compress high-mean differences, but, lacking a tie to a specific noise model, they carry no isometry interpretation and behave differently in the low-count regime.
